# Elastic Constants of Synthetic Single Crystal Corundum at Room Temperature

**DOI:** 10.6028/jres.064A.022

**Published:** 1960-06-01

**Authors:** J. B. Wachtman, W. E. Tefft, D. G. Lam, R. P. Stinchfield

## Abstract

The six elastic constants (and six elastic compliances) of corundum were determined in the kilocycle per second frequency range by an accurate resonance method. The results were checked in the megacycle per second range with a less accurate, pulse velocity method. The elastic moduli for polycrystalline alumina calculated from the single crystal compliances determined by the resonance method are in good agreement with experimental values obtained on high density polycrystalline alumina. The variation of Young’s modulus and of the shear modulus with orientation was calculated from the compliances and the results are shown graphically. The results of the present work do not agree well with previous work on single crystal sapphire. The specification of orientation and the theory used to calculate the elastic constants are given in detail to support the contention that the results of the present work are correct.

## 1. Introduction

The purpose of the present paper is to present a set of values for the elastic constants of single crystal corundum^1^ at room temperature and to argue that these values are correct despite the fact that they disagree with values reported in three other investigations. Fifty-six independent measurements on 29 different specimens yielded consistent results for the six elastic constants which this material should have.^2^

The complete set of six elastic compliances and six elastic constants were first reported by Sundara Rao [1]^3^ for synthetic sapphire. A similar determination was made by Bhimasenachar [2] using natural sapphire; very good agreement with Sundara Rao was found except for c_33_. More recently, Mayer and Hiedemann [3] have redetermined the elastic constants and compliances for synthetic sapphire with results which are rather different from the previous investigators’ values. Mayer and Hiedemann suggest possible causes of incorrect resonance frequency measurements in the experimental method used by the Indian workers.

The present work began with measurements of Young’s modulus which were intended as the basis of a study of the changes in elastic moduli and internal friction as a function of temperature. These Young’s modulus values permit calculation of four compliance constants which were found to be inconsistent with either of the previously reported sets of compliance constants. All of the previous work was done at frequencies in the megacycle per second range, but the present determination of Young’s modulus values was made in the low kilocycle per second range. A possible frequency dependence thus required consideration. The present authors undertook a broadened program with the objectives of determining a complete set of elastic constants in the kilocycle per second range and checking the results in the megacycle per second range. The results of the broadened investigation are reported in this paper.

In all the work discussed so far the classical theory of elasticity was used. This classical theory has been considered correct for more than 50 years and is still accepted by most scientists. Briefly, it assumes a linear relationship (Hooke’s law) between 6 independent components of stress and 6 independent components of strain. The theory leads to 21 independent elastic constants for the most general nonisotropic medium such as a crystal with no symmetry, and to 6 independent constants for a crystal with the symmetry of sapphire. This theory was challenged in 1951 by Laval who asserted that there are 9 independent components of stress and 9 independent components of strain which would lead to 45 independent elastic constants for a crystal with no symmetry. This idea was developed by Laval and Le Corre, and independently by Raman and his collaborators. A good list of references is given by Joel and Wooster [4]. For the case of crystals with the symmetry of sapphire, Raman [5] asserts that there are 10 independent constants instead of 6 as in the classical theory. If this generalized theory were correct, discrepancies in reported values of elastic constants might result from analysis of data in terms of a 6 constant theory when a 10- constant theory should have been used. However, results of the present work show that the classical, 6-constant theory represents the elastic behavior of sapphire in the kilocycle frequency range quite well. All equations in this paper are based on the classical theory. Because the present results do disagree with previous work, this paper presents the theory and experimental procedure in some detail.

## 2. Specification of Crystal Orientation

The values of the elastic constants (or compliances) of a nonisotropic material depend upon the choice of coordinate system. The use of a rectangular coordinate system is conventional. For each crystalline material, the orientation of this rectangular coordinate system is usually chosen to take maximum advantage of its point group symmetry, and the use of this system is implied when elastic constants are discussed. In this section the coordinate system appropriate for sapphire (called the *x*_1_*x*_2_*x*_3_ system) is described in terms of the point group symmetry and the back reflection Laue pattern. Elastic property measurements on a crystal must be related to this coordinate system in order to interpret the results of the measurements in terms of elastic constants. The line in a specimen along which Young’s modulus, the shear modulus, or the velocity of sound was measured, will be referred to as the specimen axis (SA). It is convenient to distinguish between the opposite ends of this line and the reference end will be taken as positive (+SA).

**Figure 1:**
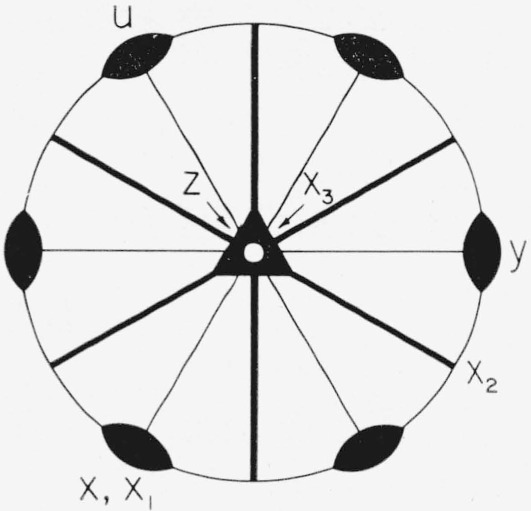
Stereogram of point group $\bar{3}$ m. The heavy lines indicate mirror planes, the open triangle indicates the $\bar{3}$ axis, and the diad symbols indicate 2-fold axes. *x*_1_*x*_2_*x*_3_ is the right-handed coordinate system used for elastic constant specification, *xyuz* is the Miller-Bravais coordinate system.

The point group of sapphire is 3*m* [6]. Figure 1 is a stereographic projection showing the symmetry elements, the rectangular system *x*_1_*x*_2_*x*_3_ to which the elastic constants are referred, and the hexagonal coordinate system *xyuz* which is used for Miller-Bravais indices. This notation for both systems is used by Nye [7] and will be used throughout this paper to refer to these coordinate systems.

**Figure 2:**
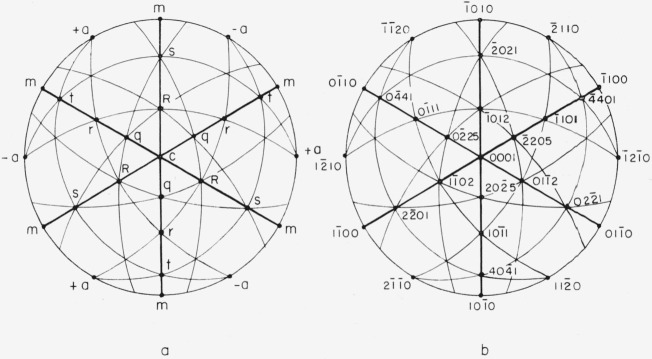
Stereograms of poles of prominent planes in sapphire. Figure 2a shows the letter symbols which can be assigned by measuring the angles between poles. Figure 2b gives the Miller-Bravais indices which require a choice of coordinate system.

Figure 2 shows a stereographic projection of prominent poles in a back reflection Laue pattern of sapphire. Figure 2a shows the letter symbols of morphological crystallography [8] which can be assigned by measuring the angles between poles. This process is without ambiguity and is described in section 6. Figure 2b shows the Miller-Bravais indices for the conventional choice of coordinate system. Figure 2a is based on angular values obtained from Winchell [8]. The recent X-ray pattern by Swanson and Fuyat [9] gives *c/a*= 1.365 for the morphological unit cell. The distinction between the morphological and the structural cells is discussed by Kronberg [10].

**Figure 3:**
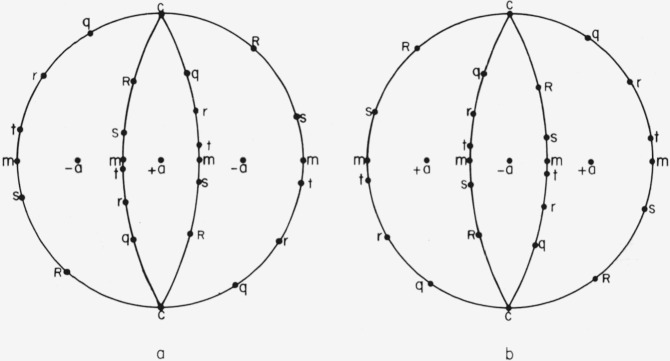
Distinction between +*a* and −*a*. The stereogram in figure 3a is centered on +*a* and shows that the sequence of poles *m t r q c R s* in the nearer mirror planes has counterclockwise sense. Figure 3b, centered on *−a*, shows that the corresponding sequence has clockwise sense.

**Figure 4:**
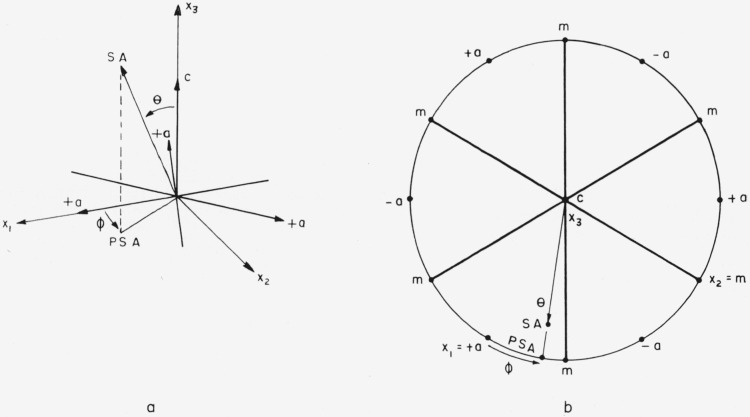
The angles used in specifying the direction of the specimen axis, SA, and its projection, PSA, in the x_1_x_2_ plane. 4a shows a perspective of the important orientation angles. 4b shows a stereographic representation of the orientation angles.

**Figure 5:**
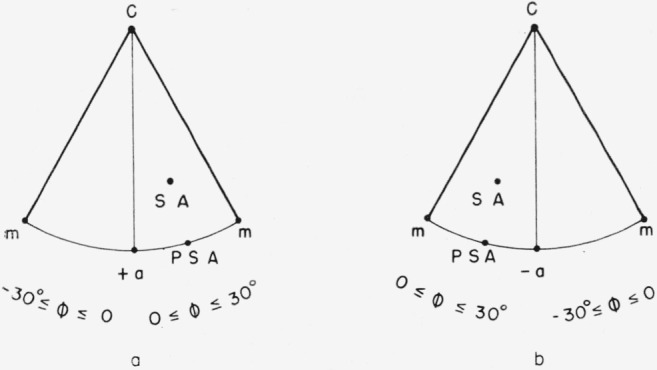
Stereograms illustrating the sign convention for *ϕ*. Note that the position of SA must be considered. The sign of *ϕ* cannot be determined from the position of PSA alone.

The *x*_1_*x*_2_*x*_3_ system is not uniquely defined by figure 1 alone. Any 1 of 6 directions might be chosen for +*x*_1_; i.e., +*x*_1_ might be chosen in either direction along any of the three 2-fold axes. However, opposite ends of a 2-fold axis can be distinguished even though point group 3*m* has a center of symmetry. The opposite ends are designated +*a* and −*a* in figure 2a. The distinction between +*a* and −*a* is illustrated in figure 3. The three +*a* directions can be identified by examining the neighboring points on the stereographic projection. The choice of which is to be called +*a* and which −*a* is, of course, a convention. This choice is important because the sign of the elastic compliance *s*_14_ depends upon it.^4^ No convention for this choice for a nonpiezoelectric crystal is given by Nye [7] or by the *Standards on Piezoelectric Crystals* [11]. The convention used here has been chosen to agree with that implied by figure 151 from Phillips [12], by figure 2 from Kronberg [10], and by figure 5 and table 1 from Winchell [8]. The angles specifying the orientation of the specimen axis are shown in figure 4. Theta, the colatitude, or zenith angle, is the angle between +*x*_3_ and SA. Phi is the angle in the *x*_1_*x*_2_ plane from +*x*_1_ to the projected specimen axis (PSA). The angle θ is exactly the same as the angle *ρ* used by Winchell [8]. However, his angle *ϕ* is measured from −*a* instead of from +*a* and is measured clockwise (looking in the −***x*_3_** direction). Winchell’s definition of *ϕ* is not used here because we wish to adhere to the usual spherical polar angles in a right-handed system.

In discussing the range of *θ* and *ϕ* it is convenient to introduce the concept of the asymmetric triangle, which is the largest spherical triangle on a stereogram not containing any symmetry related directions. Any direction outside this triangle has a symmetry related direction within it.

The range 0° ≤ *θ* ≤ 90° and —30° ≤ *ϕ*≤ 30° is sufficient to specify the orientation. That is, on a stereographic projection the range 0° ≤ *θ* ≤ 90° and −30° ≤ *ϕ* ≤ 30° defines an asymmetric spherical triangle. However, this range of *ϕ* is not sufficient to distinguish between equivalent points with mirror-image environment. For example, the environment of (11 $\bar{2}$0) is the mirror image of the environment of (2 $\bar{1}$ $\bar{1}$0). To distinguish such directions a range of 0° ≤ *θ* ≤ 90° and −30° ≤ *ϕ* ≤ 90° is useful, and Kebler and Rudness [13] have recommended the use of this range in specifying the orientation. The range of −30° ≤ *ϕ* ≤ +30° is adequate for specifying any physical property, such as elastic properties, which depend only on the direction of measurement and not upon the left-handed or right-handed distribution of neighboring points.

In carrying out the actual measurement of *ϕ* either of two equivalent methods may be used. In the first method *ϕ* is measured from PSA to *x*_1_ and the fact that *ϕ*=30°+*δ* is equivalent to *ϕ*=30° −*δ* is used to reduce any values larger than 30° to the range −30° ≤ *ϕ* ≤ 30°. In the second method ϕ is measured from PSA to the nearest *a*-type direction without regard to whether it is +*a* or −*a.* The rule shown in figure 5 is then used to determine the sign of *ϕ*.

## 3. Relation of Young’s Modulus and Shear Modulus to Elastic Compliances and to Orientation

The stresses *σ_i_*, and the strains, *ϵ_i_*, will be written in Nye’s matrix notation [7]. Hooke’s law can be written in terms of the elastic constants, *c_ij_*, as

$$\sigma_{i}=\sum_{j}c_{ij}\epsilon_{j}$$ (1)

or in terms of the elastic compliances, *s_ij_* as

$$\epsilon_{i}=\sum_{j}s_{ij}\sigma_{j}$$ (2)

where the matrix of the *s_ij_* is the inverse of the matrix of the *c_ij_*. For any material, *c_ij_=c_ji_* (and *s_ij_=s_ji_*) which reduces the number of independent constants (or compliances) to 21. For sapphire, the symmetry of point group $\bar{3}$*m* further reduces the number of independent elastic constants (or compliances) to six and the resulting matrices are shown in table 1.

In relating the elastic compliances to Young’s modulus, it is necessary to distinguish between the “free” Young’s modulus, *E_f_*, and the “pure” Young’s modulus, *E_p_*, when analyzing flexural or torsional tests. The free Young’s modulus is the value obtained when the specimen is completely free to deform elastically under the applied tensile stress. The pure Young’s modulus is the value obtained when the specimen is tested in flexure and is prevented from twisting. In an isotropic medium the free and pure moduli are identical. Calculation of Young’s modulus from flexural vibrations of long, thin rods of nonisotropic material corresponds to measurement of *E_f_.* A similar distinction must be made with the shear modulus, *G.* For rods of nonisotropic material of the dimensions used in this work, the modulus determined from torsional vibrations is very accurately the pure shear modulus, *G_p_.* The proof that *E_f_* and *G_p_* are the measured quantities involves the substitution in equations given by Hearmon [14] and by Brown [15]. We shall omit the proof here and give the results connecting the quantities determined experimentally, *E_f_* and *G_p_*, with the elastic compliances. Consider a rectangular coordinate system $x_{1}^{\prime }x_{2}^{\prime }x_{3}^{\prime }$ with the $x_{3}^{\prime }$ axis along SA. For stress application along the $x_{3}^{\prime }$ axis [14, 15]:

$$s_{33}^{\prime }=\frac{1}{E_{f}},$$ (3)

$$\frac{s_{44}^{\prime }+s_{55}^{\prime }}{2}=\frac{1}{G_{f}},$$ (4)

$$G_{f}=G_{p}(1-\epsilon^{\prime }),$$ (5)

where

$$\epsilon^{\prime }=\frac{{s^{\prime }}_{34}^{2}+{s^{\prime }}_{35}^{2}}{s_{33}^{\prime }(s_{44}^{\prime }+s_{55}^{\prime })}.$$ (6)

Equations (4), (5), and (6) can be combined to give

$$\frac{1}{G_{f}}=\frac{1}{G_{p}}+\frac{{s^{\prime }}_{34}^{2}+{s^{\prime }}_{35}^{2}}{2s_{33}^{\prime }},$$ (7)

which is a more useful relation between *G_p_* and *G_f_* than eq (5).

**Figure 6:**
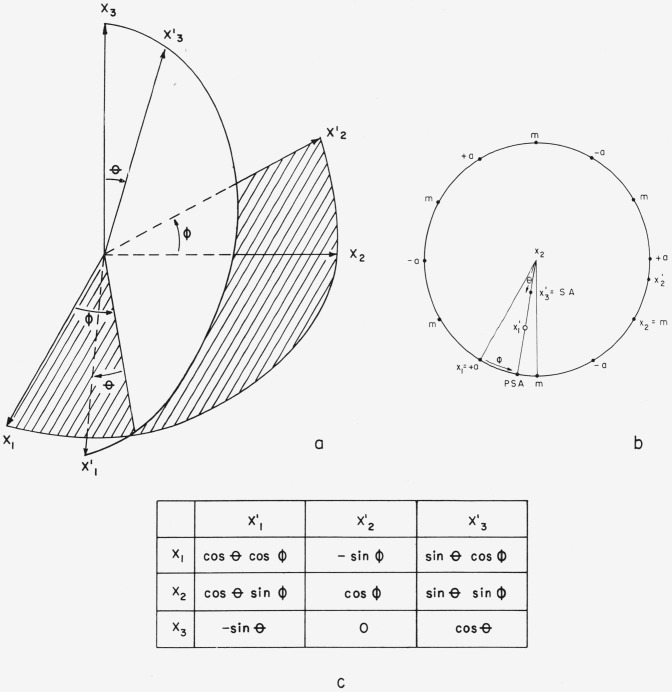
The rotated coordinate system *x*_1_*x*_2_*x*_3_ and the direction cosine scheme. Figure 6a is a perspective drawing of the relation between $x_{1}^{\prime }x_{2}^{\prime }x_{3}^{\prime }$ and *x*_1_*x*_2_*x*_3_. Figure 6b shows the relation stereographically. Figure 6c gives the values of the direction cosines.

The primed quantities in eqs (3), (4), and (7) must be related to the orientation and to the elastic compliances measured in the *x*_1_*x*_2_*x*_3_ system. It can be shown [14] that the values of $s_{33}^{\prime }$, $s_{44}^{\prime }+s_{55}^{\prime }$and ${s^{\prime }}_{34}^{2}+{s^{\prime }}_{35}^{2}$ depend only upon the direction of $x_{3}^{\prime }$ and not upon the direction of $x_{1}^{\prime }$ and $x_{2}^{\prime }$. Accordingly $x_{1}^{\prime }$ and $x_{2}^{\prime }$ were chosen to simplify the transformation equations as much as possible subject to the condition that $x_{1}^{\prime }x_{2}^{\prime }x_{3}^{\prime }$ form a right-handed rectangular set. This was accomplished by choosing $x_{2}^{\prime }$ to lie in the *x*_1_*x*_2_ plane, then choosing $x_{1}^{\prime }$ orthogonal to $x_{2}^{\prime }$ and $x_{3}^{\prime }$. The relation of $x_{1}^{\prime }x_{2}^{\prime }x_{3}^{\prime }$ to *x*_1_*x*_2_*x*_3_ is shown in figure 6.

Equation 25 on page 70 and equation 10 on page 61 of Cady [16] give the general transformation for $s_{33}^{\prime }$ and $s_{44}^{\prime }+s_{55}^{\prime }$ respectively. An error in sign was found in 12 of the last 18 terms of the equation for $s_{44}^{\prime }+s_{55}^{\prime }$. When this error is corrected, the resulting expressions are:

$$\begin{array}{l} s_{33}^{\prime }=\frac{1}{E_{f}(\theta ,\phi )}=s_{11}\;\sin{\;}^{4}\theta +s_{33}\;\cos{\;}^{4}\theta \\ \;+(2s_{13}+s_{44})\;\sin{\;}^{2}\theta \;\cos{\;}^{2}\theta \\ \;+2s_{14}\;\sin{\;}^{3}\theta \;\cos\theta \;\sin3\phi , \end{array}$$ (8)

and

$$\begin{array}{c} \frac{s_{44}^{\prime }+s_{55}^{\prime }}{2}=\frac{1}{G_{f}(\theta ,\phi )}=(s_{11}-s_{12})\;\sin{\;}^{2}\theta +s_{44}\left(\frac{1+\cos{\;}^{2}\theta }{2}\right)\\ \;+2(s_{11}+s_{33}-2s_{13}-s_{44})\;\sin{\;}^{2}\theta \;\cos{\;}^{2}\theta \\ -4s_{14}\;\sin{\;}^{3}\theta \;\cos\theta \;\sin3\phi , \end{array}$$ (9)

which agree with eqs 48 and 49, page 76 of Cady [16] for trigonal symmetry.

The transformations for $s_{34}^{\prime }$ and $s_{35}^{\prime }$ were obtained by using the general transformation law for fourth rank tensors, then reducing this to the trigonal symmetry of sapphire and making use of the particular choice of $x_{1}^{\prime }x_{2}^{\prime }$described above. The resulting expressions are

$$s_{34}^{\prime }=3s_{14}\;\sin{\;}^{2}\theta \;\cos\theta \;\cos3\phi ,$$ (10)

and

$$\begin{array}{c} s_{35}^{\prime }=2(2s_{13}+s_{44}-s_{11}-s_{33})\;\sin\theta \;\cos{\;}^{3}\theta \\ +2\left(s_{11}-s_{13}-\frac{s_{44}}{2}\right)\;\sin\theta \;\cos\theta \\ s_{14}\;\sin\theta \;\sin3\theta \;\sin3\phi . \end{array}$$ (11)

A set of elastic compliances can be determined from these equations in the following manner: It is necessary to determine the values of *E_f_* and *G_p_* on at least four rods of known orientation. Equation (8) is written for each rod and the resulting system of simultaneous linear equations is solved for *s*_11_, *s*_33_, 2*s*_13_+*s*_44_, and *s*_14_. These values are substituted into eqs (8), (10), and (11) and the results are used in eq (7) to calculate *G_f_* for each rod. These values of *G_f_*, *θ*, and *ϕ* are substituted into eq (9) for each rod. The resulting system of simultaneous linear equations is solved for *s*_11_−*s*_12_, *s*_44_, *s*_11_+*s*_33_−2*s*_13_−*s*_44_, and *s*_14_

## 4. Relation of Elastic Moduli to Resonance Frequencies

The values of *E_f_* and *G_p_* needed for the calculation of the elastic compliances were obtained by resonance frequency measurements on thin, circular rods. Young’s modulus was calculated from the longitudinal resonance frequency and independently from the flexural resonance frequency. The shear modulus was calculated from the torsional resonance frequency. For both of the Young’s modulus calculations, the equation relating the resonance frequency to the appropriate modulus is approximate, but the approximations are very good.

For longitudinal vibrations, Young’s modulus of an isotropic medium can be calculated from Rayleigh’s equation [17] which can be written

$$E=4\rho l^{2}f^{2}{\left(1+\frac{\pi^{2}\sigma^{2}r^{2}}{4l^{2}}\right)}^{2},$$ (12)

where *ρ* is the density in grams per cubic centimeter, *l* is the length in centimeters, *f* is the longitudinal resonance frequency in cycles per second, *σ* is Poisson’s ratio, *r* is the radius in centimeters, and *E* is Young’s modulus in dynes per square centimeter; 1 dyne/cm^2^=10^−9^ kilobars= 1.020×10^−6^ kg/cm^2^= 1.450×10^−5^ lb/in^2^. The term in parenthesis is the Rayleigh correction term for the finite thickness of the rod and neglects higher powers of *r/l.* Bancroft [18] has made an accurate calculation of the velocity of longitudinal waves as a function of *r*/λ, where λ is the wavelength which is equal to 2*l* for resonance in the fundamental mode. The smallest value for which Bancroft gives a numerical result corresponds to *r/l* = 0.05. Assuming Poisson’s ratio is 0.25, the Rayleigh correction for this value is 1.00077 and Bancroft’s results give 1.00078. Evidently the Rayleigh correction is very good for small values of *r*/*l*. The largest value of *r*/*l* used in the present work was 0.0125 so that eq (12) is thought to be very accurate. For the range of *r*/*l* used in this work, the entire correction factor can be taken to be 1.0000. This equation was derived on the assumption of elastic isotropy, but the actual specimens were anisotropic. Equation (12) gives *E_f_* for crystalline rods of the dimensions used. The value of Poisson’s ratio varies somewhat for single crystals but for poly crystalline alumina is about 0.25, and since this only enters in the correction term, it seems that eq (12) can be used with this value of Poisson’s ratio.

For flexural vibrations of an isotropic medium the best existing theory seems to be that based on an approximate differential equation derived by Timoshenko [19] and studied by Goens [20] and by Pickett [21]. Pickett’s results are given in the form of a correction factor, *T*, which multiplies the result for an infinitely thin rod. His results can be expressed as

$$E=0.31547\frac{\rho l^{4}f^{2}}{r^{2}}T,$$ (13)

where *E* is in dynes per square centimeter, and the symbols have the same meaning as in eq (12) except *f* which here is the flexural resonance frequency. In his original paper, Pickett [21] gives equations for calculating *T* for Poisson’s ratio values of zero, 1/6, and 1/3. A subsequent ASTM Standard [22] gives an interpolation formula without giving the derivation or stating the range of validity. It seems best to use Pickett’s original formula and interpolate using a quadratic approximation. The question is not significant for rods of the size used in this investigation, the thinnest rods having ***r***=0.050 In. (*r*/*l*=0.0083) and *T*= 1.0015, the thickest rods having *r*=0.075 in. (*r/l*=0.0125) and *T*=1.0033.

The equation relating the shear modulus to the resonance frequency of an isotropic cylinder can be derived by considering the propagation of torsional waves and requiring that the wavelength have a value such that standing waves are formed. The result is

$$G=4\rho l^{2}f^{2},$$ (14)

where *G* is the shear modulus in dynes per square centimeter, *f* is the torsional resonance frequency, *ρ*is the density in grams per square centimeter, and *l* is the length. The significance of this equation for crystalline cylinders has been considered by Brown [15] and by Hearmon [14]. The details are complicated, but the result is, as previously stated, that for thin rods eq (14) gives *G_p_* and eq (13) gives *E_f_*.

## 5. Relation of Elastic Constants to the Velocity of Ultrasonic Waves

The theory required for the determination of the elastic constants of sapphire from resonance frequency measurement was given in sections 3 and 4. The present section presents the theory required for elastic constant determinations from wave velocity measurements. The velocity of sound as a function of specimen size has been studied by Tu, Brennan, and Sauer [23]. They found that in cylindrical rods the velocity of longitudinal waves depended upon *r*/λ where *r* is the radius of the rod and λ is the wavelength of the sound wave. For values of *r*/λ larger than 2.5, the longitudinal waves traveled at the speed of plane waves in an infinite medium.

Similar results should hold for waves propagated in a rectangular block. The longest wavelength used in the present work was about 0.11-cm and the smallest block was 1.25-cm thick. These values give a thickness to wavelength ratio of 11.4. It seems that the theory for plane waves in an infinite medium can therefore be used.

This theory is summarized by Kolsky [24] and by Markham [25]. The results are contained in a cubic equation in the variable *x=ρv*^2^ where *ρ* is the density and *v* is the velocity. This equation can conveniently be written in determinant form

$$\left|\begin{array}{ccc} A-x & H & G\\ H & B-x & F\\ G & F & C-x \end{array}\right|=0.$$ (15)

where

$$A=c_{11}l^{2}+c_{66}m^{2}+c_{55}n^{2}+2c_{16}lm+2c_{56}mn+2c_{15}nl,$$ (16)

$$B=c_{66}l^{2}+c_{22}m^{2}+c_{44}n^{2}+2c_{26}lm+2c_{24}mn+2c_{46}nl,$$ (17)

$$C=c_{55}l^{2}+c_{44}m^{2}+c_{33}n^{2}+2c_{45}lm+2c_{34}mn+2c_{35}nl,$$ (18)

$$F=c_{56}l^{2}+c_{24}m^{2}+c_{34}n^{2}+(c_{25}+c_{46})lm+(c_{23}+c_{44})mn+(c_{36}+c_{45})nl,$$ (19)

$$G=c_{15}l^{2}+c_{46}m^{2}+c_{35}n^{2}+(c_{14}+c_{56})lm+(c_{36}+c_{45})mn+(c_{13}+c_{55})nl,$$ (20)

and

$$H=c_{16}l^{2}+c_{26}m^{2}+c_{45}n^{2}+(c_{12}+c_{66})lm+(c_{25}+c_{46})mn+(c_{14}+c_{56})nl.$$ (21)

In these equations the *c_ij_* are the elastic constants and *l, m, n* are the direction cosines of the normal to the wave front. The distinction between velocity surface and wave surface for elastic waves has been discussed by Musgrave [26] and Markham [25]. It is sufficient to state that the measured velocities in the direction defined by *lmn* are those given by eq (15). It is important to notice that both the elastic constants and the direction cosines are referred to the same coordinate system. For sapphire, if the coordinate system is chosen to be the *x*_1_*x*_2_*x*_3_ system previously described, the matrix of elastic constants given in table 1 may be substituted to simplify eqs (16) through (21). This simplification has been done by Bhimasenachar [27] for calcite which has the same point group and consequently the same elastic constant matrix as sapphire. The resulting equations can be expressed directly in terms of the orientation by substituting *l*=sin*θ*cos*ϕ*,*m*=sin*θ*sin*ϕ*, and *n*=cos*θ.* These substitutions give

$$\begin{array}{c} A=c_{11}\;\sin^{2}\theta \;\cos^{2}\phi +\frac{1}{2}(c_{11}-c_{12})\;\sin^{2}\theta \;\sin^{2}\phi +\\ \;c_{44}\;\cos^{2}\theta +2c_{14}\;\sin\theta \;\cos\theta \;\sin\phi , \end{array}$$ (22)

$$\begin{array}{c} B=\frac{1}{2}(c_{11}-c_{12})\;\sin^{2}\theta \;\cos^{2}\phi +c_{11}\sin^{2}\theta \;\sin^{2}\phi \\ +c_{44}\;\cos^{2}\theta -2c_{14}\;\sin\theta \;\cos\theta \;\sin\phi , \end{array}$$ (23)

$$C=c_{44}\;\sin^{2}\theta +c_{33}\;\cos^{2}\theta ,$$ (24)

$$F=c_{14}\;\sin^{2}\theta (1-2\;\sin^{2}\phi )+(c_{13}+c_{44})\;\sin\theta \;\cos\theta \;\sin\phi ,$$ (25)

$$G=2c_{14}\;\sin^{2}\theta \;\sin\phi \;\cos\phi +(c_{13}+c_{44})\;\sin\theta \;\cos\theta \;\cos\phi ,$$ (26)

and

$$H=\frac{1}{2}(c_{11}+c_{12})\;\sin^{2}\theta \;\sin\phi \;\cos\phi +2c_{14}\;\sin\theta \;\cos\theta \;\cos\phi .$$ (27)

To solve for the velocity of sound in a given direction, the direction cosines must be substituted into the expressions for *A* … *H* which must then be substituted into (15) and the resulting cubic equation solved. For some directions this calculation is easy. For example, waves propagated along the $\bar{3}$axis will have *l*=0, *m*=0, *n*= 1.

Equation (15) reduces to

$$\left|\begin{array}{ccc} c_{44}-x & 0 & 0\\ 0 & c_{44}-x & 0\\ 0 & 0 & c_{33}-x \end{array}\right|=0$$ (28)

which gives three roots

$$\rho v^{2}=c_{33},\;c_{44},\;c_{44}.$$ (29)

These roots correspond to the propagation of a longitudinal wave with velocity $\sqrt{c_{33}/\rho }$ and two transverse waves with velocity $\sqrt{c_{44}/\rho }$. For other directions eq (15) does not reduce to such a simple form and the waves are quasi-longitudinal and quasi-transverse rather than truly longitudinal and transverse.

A simplified method of treating directions for which eq (15) is difficult to solve has been used by each of the previous workers on the elastic constants of sapphire. The method has been most fully described by Mayer and Hiedemann [3]. They assert that for any direction the velocity of the quasilongitudinal and quasi-transverse waves are given respectively by

$$\rho v_{L}^{2}=c_{33}^{\prime }$$ (30)

and

$$\rho v_{S}^{2}=c_{44}^{\prime }$$ (31)

where $c_{33}^{\prime }$ and $c_{44}^{\prime }$ are the value of *c*_33_ and *c*_44_ when the coordinate system is transformed into one with the $x_{3}^{\prime }$ axis in the direction of propagation. Expressions for $c_{33}^{\prime }$ and $c_{44}^{\prime }$are given by Mayer and Hiedemann [3]. The present writers do not question the transformation equations for $c_{33}^{\prime }$ and $c_{44}^{\prime }$ but do assert that eqs (30) and (31) are not true in general. That is, $\rho v_{L}^{2}$ or $\rho v_{S}^{2}$ will in general be a more complicated function of the elastic constants and direction of propagation which may reduce to eqs (30) and (31) for certain directions of propagation.

An example of a direction for which the simplified method gives a different answer than the general eq (15) is the *x*_1_ direction (parallel to a 2-fold axis) in sapphire. Using eqs (30) and (31) Mayer and Hiedemann obtain for *ρv*^2^ the three values

$$\rho v_{L}^{2}=c_{11},$$ (32)

$$\rho v_{S}^{2}=c_{44},$$ (33)

and

$$\rho v_{S}^{2}=c_{66}=\frac{1}{2}(c_{11}-c_{12})$$ (34)

Actually substituting into eq (15) gives

$$\rho v_{L}^{2}=c_{11},$$ (35)

$$\rho v_{S}^{2}=\frac{1}{2}(c_{66}+c_{44})\pm \frac{1}{2}(c_{66}-c_{44})\times \sqrt{1+\frac{4c_{14}^{2}}{{\left(c_{66}-c_{44}\right)}^{2}}}.$$ (36)

The results agree for the longitudinal wave but do not agree for the transverse wave unless *c*_14_ is zero. In practice it might happen that $4c_{14}^{2}/{\left(c_{66}-c_{44}\right)}^{2}$ is small compared with one. If this were so, eqs (30) and (31) would give a good approximation for this particular direction but might not for a different direction. It seems that the results calculated from (30) and (31) would have to be checked against the results obtained from the fundamental eq (15), for each direction of propagation. This fact, of course, would mean that no time would be saved by the use of the simplified method. In the present work, only the exact eq (15) was used.

It is perhaps worth noting why the simplified method should not be expected to be correct. If the *x*_1_*x*_2_*x*_3_ coordinate system is transformed into the $x_{1}^{\prime }x_{2}^{\prime }x_{3}^{\prime }$ system, every term in eq (15) can be transformed to give a correct result. Equation (28), however, is a special case of (15) resulting from a special choice of coordinate system and direction of propagation. If the nonzero terms are transformed by a rotation of the coordinate system, eqs (30) and (31) will result, but these equations cannot be expected to hold in general for directions of propagation other than the direction assumed in deriving eq (28).

The error in the simplified method can perhaps be more easily seen from a physical argument. The transformed constants $c_{33}^{\prime }$, $c_{44}^{\prime }$, and $c_{55}^{\prime }$, would be appropriate for calculation of the velocities of waves traveling in the $x_{3}^{\prime }$ direction and with particle motion along the $x_{3}^{\prime }$, $x_{2}^{\prime }$, and $x_{1}^{\prime }$ axes, respectively. However, as Cady [16] has shown, these are not necessarily the directions of motion of the particles, and the deviation in some cases can be quite large.

A method of solving eq (15) for the elastic constants which utilizes tensor transformations has been developed by Neighbours [28]. In his method all terms are taken into account, and in place of eqs (30) and (31) a set of equations is obtained each of which contains an infinite series which presumably converges rapidly. Equations (30) and (31) are equivalent to the zeroth approximation in this perturbation method of Neighbours. He shows how rapidly the approximations converge in some cases but does not discuss the trigonal system.

In his paper on calcite, Bhimasenachar [27] uses equations equivalent to (30) and (31) but explicitly states that small coupling terms have been neglected. In his subsequent paper on sapphire, Bhimasenachar [2] uses the simplified method without mentioning that it is approximate. Both Sundara Rao [1] and Mayer and Hiedemann [3] also use the simplified method but present it as if it were exact.

## 6. Specimens and Experimental Procedure

**Figure 7:**
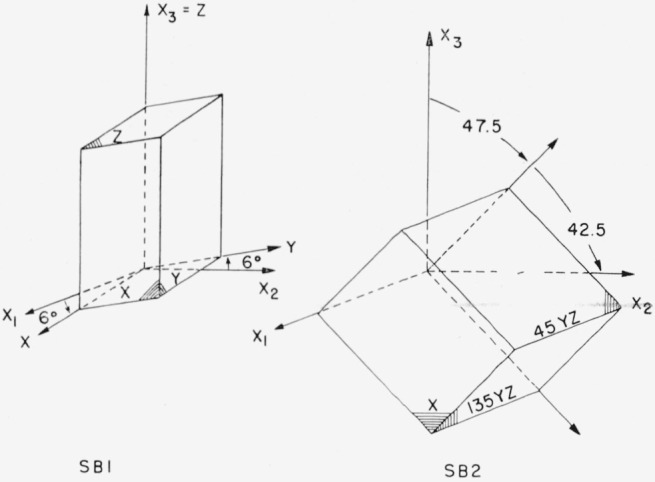
The orientation of the two rectangular block specimens.

The single crystal sapphire specimens were of two types: rods and blocks. The dimensions, mass, surface condition, and orientation of the 29 rod specimens are described in table 2. Two rectangular block specimens were ground to our order by the Linde Company. The first block, SB1, was supposed to be 1 ×½× ½ in. with the long axis parallel to the *x_3_* axis and one face perpendicular to the *x*_1_ axis. The second block, SB2, was specified as 1 × ¾ *×* ¾ in. with the long axis in the *x*_2_*x*_3_ plane making an angle of 135° with *x*_3_ and with one face perpendicular to *x*_1_. The actual orientations are shown in figure 7. It will be seen that for SB1 the *θ* values are correct, but *ϕ* is 6° instead of zero. For SB2 the *ϕ* values are correct, but *θ* is 47.5 instead of 45.0°.

A Laue camera of conventional design was used to determine the orientation; 35KVp X-radiation (copper target) was used with exposure times of about 6 to 10 hr. The samples were supported with the specimen axis perpendicular to the X-ray beam and parallel to the edge of a small shield on the film holder. The shadow cast by this shield on the film established the reference line on the film. A specimen to film distance of 3 cm was used, and the stereographic projection was made according to the procedure described by Barrett [29] using the Greninger chart given in his figure 22 on page 170, and a 21-in. Wulff net. The resulting projection was then indexed with the letter symbols shown in figure 2a. This indexing was done by a trial and error method of making a tentative assignment and comparing angles with the values for sapphire given by Winchell [8]. This process was continued until the proper fit was obtained.

**Figure 8:**
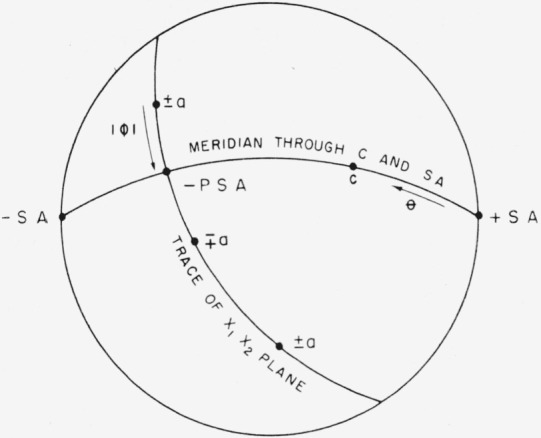
Stereogram illustrating the actual measurement of ϕ and θ.

The second method of measuring *ϕ*, described in section 2, was used. The actual construction obtained from a back reflection Laue pattern always resembled the projection shown in figure 8, that is, the opposite ends of the specimen axis were at opposite points of the stereographic equator. The value of *θ* can be read directly from this projection, as shown, but the specimen axis must be projected on the *x*_1_*x*_2_ plane to measure *ϕ*. Projection of +SA would require rotation of the whole pattern because PSA would lie on the hemisphere not shown in figure 8. This projection can be done but requires more graphical construction with attendant possibility of error. It is more convenient to project −SA and choose *x*_1_ as the ± *a* direction nearest −PSA. The sign convention for determining is shown in figure 5. Orientations were determined twice on 12 specimens with the resulting standard deviations Δ*θ* = 0.6° and Δ*ϕ* = 1.6°.

The longitudinal resonance frequency of each rod shaped specimen was measured by hanging the specimen vertically from a crystal pickup. A single, fine cotton thread was tied around the specimen somewhat off center so that the specimen hung approximately vertically. The other end of the thread was tied to the needle of a high output Rochelle salt pickup. Vibration was excited by placing a high frequency speaker just under the specimen and driving through air. The electronic components used to drive the speaker, to amplify the pickup signal, and to measure the frequency have been described [30]. With this system it was possible to detect resonances in the range 30 to 50 kc. The response was weak but very sharp, and careful hunting was sometimes necessary.

The flexural resonance frequency was measured with the specimen suspended horizontally on two threads near the nodes. One thread was driven by a magnetic cutting head. The other thread was connected to a crystal pickup. Each specimen would vibrate in two flexural modes at right angles to each other. By turning the specimen in the string it was possible to excite either mode at will and to determine the direction of vibration for each. The two resonance frequencies usually differed by a few cycles, presumably because of small variations in the diameter of the specimen. The average of the two resonance frequencies was used in eq (13). An average value of *r*^2^, for each rod calculated from the density, the mass, and the length, was used. This approach was used because the density could be determined accurately by hydrostatic weighing, but the average radius was more difficult to measure accurately.

The torsional resonance frequency was determined with the same speaker and pickup used in the longitudinal resonance frequency measurements. The tweeter was modified by covering its output opening with a sheet of paper. The thread used to drive the specimen was fastened to the center of this sheet. The thread was wrapped around the specimen several times to provide an off-center driving force and the pickup thread was wrapped around the specimen in the opposite direction. Careful hunting was again necessary to find the weak but sharp resonance. It was necessary to distinguish between the fundamental of torsion and the overtones of flexure. This distinction was accomplished by moving the driving and pickup strings until the nodes were located. The counter used for frequency measurements had an accuracy of ± 0.1 cps.

The velocity of sound measurements on the two block specimens were made by sending an ultrasonic pulse into the specimen and measuring the transit time. A quartz crystal 0.5 in. in diameter and cut to resonate at 10 Mc was used as transmitter and pickup. An X-cut crystal was used to generate longitudinal waves, and an AC-cut crystal was used for transverse waves. These crystal cuts are described by Buchanan [31]. The crystals were mounted on the specimen with salol (phenyl salicylate). The specimen was coated with a very thin layer of evaporated aluminum before the crystals were mounted in order to provide a ground.

The crystals were excited and the echoes were amplified with a commercial ultrasonic generator and receiver. This unit had a pulsed oscillator of variable frequency with a fixed pulse repetition rate of 100/sec. The amplified output of the oscillator was connected to an electrode on a quartz crystal. The same electrode served as an input connector for the receiver. The output of the receiver was connected to one input of an oscilloscope with a dual trace preamplifier. A crystal controlled 1–Mc oscillator was connected to the other input of the dual trace preamplifier. The oscilloscope thus displayed the standard 1–Mc signal on one trace followed by the echo pattern on the next trace. These two traces were simultaneously visible, and the 1–Mc pattern was used as a time scale for the transit time measurement. The general technique of pulse velocity measurement is reviewed by Huntington [32].

The sweep expansion feature of the oscilloscope could be read to 0.01 *μ* sec. The actual measurements were much less accurate, however, because the pulses were rounded and did not have a sharply defined leading edge. Consequently the maximum of the pulse envelope was used as a reference point. Repeated measurements suggested that the resulting transit time might have an error of several percent. For this reason no transit time correction, such as that discussed by Overton and Gaffney [33], was made. The measured velocities given in this report should be examined with this in mind. They are provided as a rough check on the accurate values determined by the resonance methods to show that the values of the elastic constants, within the accuracy of pulse velocity measurements, show no frequency dependence.

## 7. Results

The resonance frequencies are summarized in table 3. These values were used to calculate *E_f_* and *G_p_* using the equations of section 4. The method described in section 3 was then used to calculate the elastic compliances, *s_ij_.* Tables 3 through 7 show various stages in these calculations and indicate the precision. The reciprocals of the *E_f_* values used in solving eq (8) are given in table 4, and the resulting values of the compliance combinations are given in the first half of table 6. The difference column in table 4 shows how well the observations on all 29 rods were fitted by the four parameters of eq (8). The shear modulus calculations are summarized in table 5, and the resulting compliance combinations are given in the second half of table 6. The difference column of table 5 shows how accurately eq (9) fitted the data.

The final result for the elastic compliances and constants is given in table 7. The six compliances were calculated from the eight values in table 6 by weighting each value according to its standard deviation. The constants were calculated from the compliances using the standard matrix inversion equations given, for example, by Nye [7].

**Figure 9:**
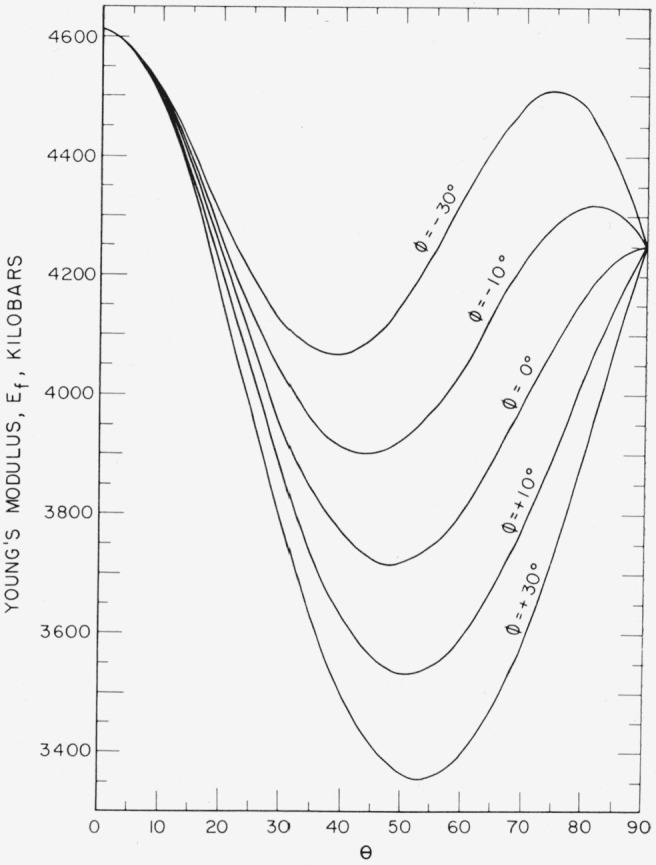
Young’s modulus, *E_f_*, as a function of orientation for single crustal sapphire.

The *c_ij_* values of table 7 were used in eq (15) to calculate the velocity of sound for comparison with the measured velocities for all directions of propagation normal to the faces of SB1 and SB2. In some cases this necessitated the solution of a cubic equation by successive approximations. The results are given in table 8 along with the measured values. In this table the faces have been described by the labels shown in figure 9, e.g., the 45YZ face. The actual orientation was used in the calculations, however; that is, the 45YZ face normal actually had *θ* = 47.50° instead of 45°.

**Figure 10:**
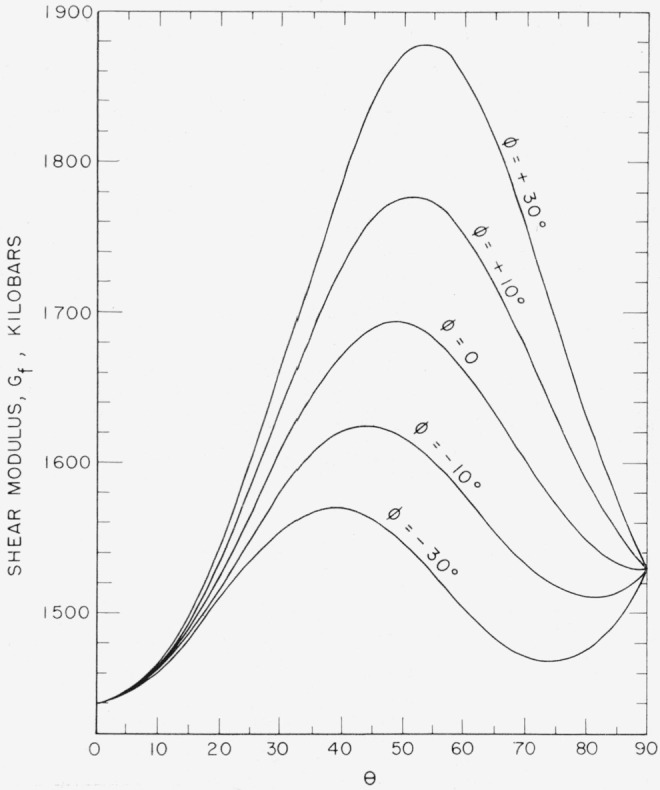
The shear modulus, *G_f_*, as a function of orientation for single crystal sapphire.

**Figure 11:**
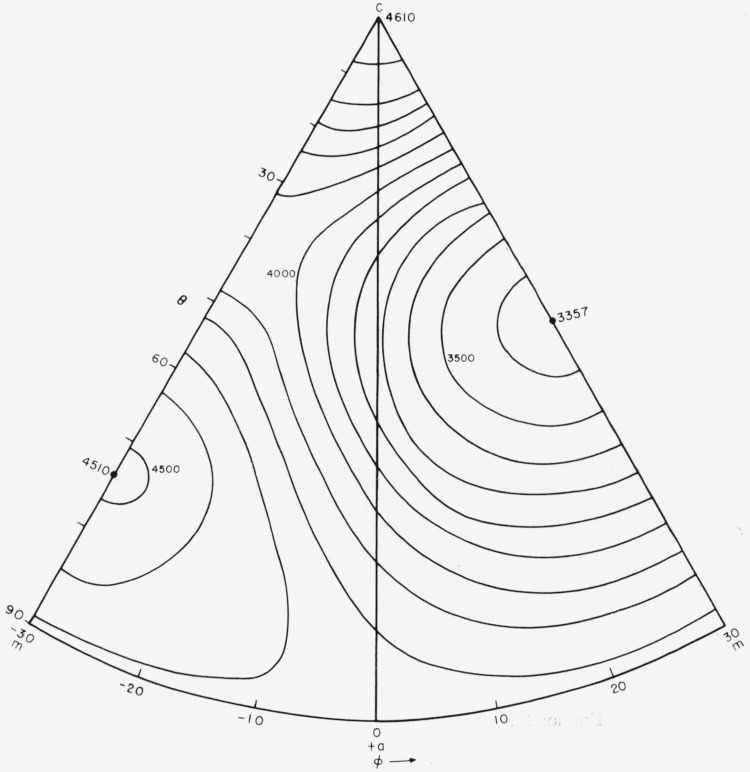
Young’s modulus, *E_f_*, intervals of 100 kilobars.

**Figure 12:**
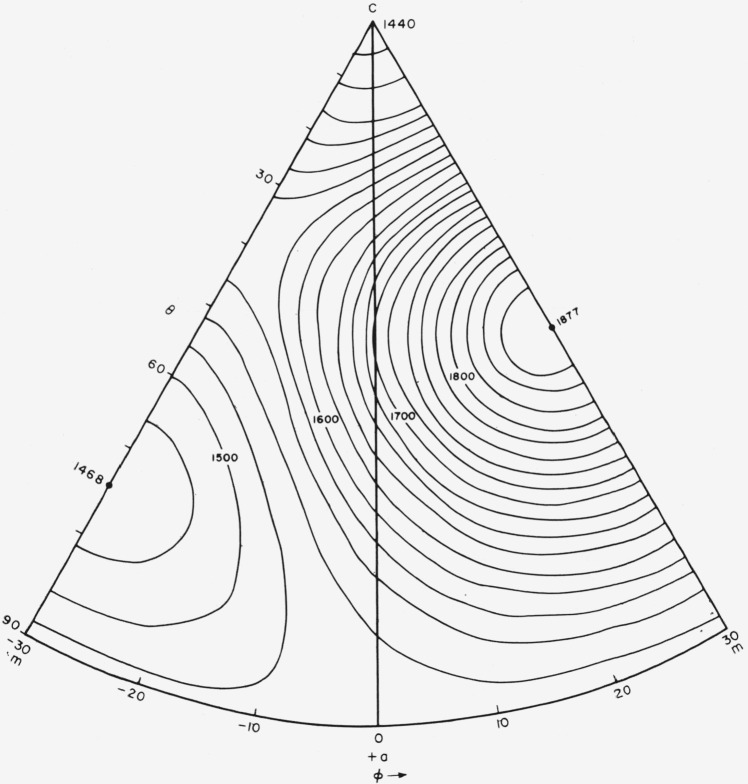
Shear modulus, *G_f_*, intervals of 20 kilobars.

The *s_ij_* values were used to calculate *E_f_* and *G_f_* as a function of orientation. The results are shown in figures 9 and 10 and in contour form in figures 11 and 12.

## 8. Discussion

The values of the elastic constants and compliances for sapphire which have been reported by other investigators are given in table 7. Mayer and Hiedemann have suggested that the method of exciting vibrations used by the Indian workers may give spurious resonances. For this reason we compare primarily with the set of values given by Mayer and Hiedemann.

The method used by Mayer and Hiedemann gave the *c_ij_* values directly; an error in a single one of the *c_ij_* might lead to errors in several of the *s_ij_* values. Accordingly we compare *c* values rather than *s* values. *c*_11_ and *c*_33_ are in good agreement, but the other values are not. The difference in sign of *c*_14_ is striking. As was pointed out earlier, the sign of *c*_14_ (and *s*_14_) depends on which end of the 2-fold axis is taken as +*x*_1_. The conventions given by Nye [7] and by the *Standards on Piezoelectric Crystals* [11] leave this unspecified. This point is not mentioned by any of the previous workers on sapphire, and we have not been able to find any information in their papers on which choice was made. If Mayer and Hiedemann used the opposite end of a 2-fold axis as +*x*_1_, the difference in sign of *c*_14_ would be accounted for and the values of *c*_14_ would be in fair agreement. The most troublesome disagreement is that between the value of *c*_44_ obtained in the present work, 1.47×10^12^ dyne/cm^2^, and the value of 2.06×10^12^ dyne/cm^2^ obtained by Mayer and Hiedemann. The 1.47 value cannot be very much in error because the value of the velocity of the transverse wave in the *x*_3_ direction depends only on this constant, and the measured value is −2.3 percent from the calculated value as shown in table 8. The fact that eqs (30) and (31), used by Mayer and Hiedemann, are not strictly correct was pointed out in section 5. However, it does not seem that this fact has any connection with the disagreement in the values of *c*_44_ because these equations are not needed for a wave propagated in the *x*_3_ direction.^4^

A comparison can be made to support the validity of the present data. Lang [34] has reported values of elastic moduli determined dynamically on hot pressed polycrystalline alpha alumina of density 3.983 g/cm^3^. This density is so close to the value of 3.986 g/cm^3^ obtained on single crystals that the values of the elastic moduli of his specimens must be only slightly less than the values for zero porosity. He gives *E*=4012 kilobars and *G*=1591 kilobars as his best values. An exact theoretical relationship between single crystal elastic constants and polycrystalline moduli has not been developed, but two good approximations are discussed by Huntington [32]. One method, due to Voigt, should give values slightly too high, and the other method, due to Reuss, should give values slightly too low. Using the elastic constant and compliance data of table 7 and the equations given in Huntington’s article, one obtains the results shown in table 9. The values of *E* and *G* calculated from the elastic constants obtained in this investigation are in rather good agreement with Lang’s values.

All elastic constants, compliances, and moduli so far discussed have been for adiabatic conditions; i.e., measured under conditions when appreciable heat flow could not occur during one cycle of vibration. Isothermal values are measured in static tests. Hearmon [14] gives an equation from which the difference between isothermal and adiabatic values can be calculated. This difference ranges from 0.2 percent for *s*_11_ to 1 percent for *s*_13_.

The compressibilities can be calculated from the compliances using equations given by Nye [7]. The adiabatic compressibilities calculated in this way are given in table 10. These should differ from the isothermal compressibilities by about 1 percent. Values of the isothermal compressibilities measured in static tests by Bridgman [35] and by Madelung and Fuchs [36] are given in table 10. Bridgman’s linear compressibility parallel to the *x*_3_ axis is based on the initial slope of his curve and is inconsistent with all the other data. It is listed in the first row of measured values in table 10 even though it does not appear to be consistent with his reported value of volume compressibility. If the slope of the subsequent section of Bridgman’s curve is used, the values labeled “Bridgman, interpreted” are obtained. The present work is in somewhat better agreement with the experimental values of volume compressibility than the results of Mayer and Hiedemann. Visual inspection of Bridgman’s curves suggests that the difference between the value of 3.6×10^−13^ cm^2^/dyne, calculated from his data, and 3.988×10^−13^ cm^2^/dyne may be largely due to experimental error in his data.

Finally, the difference columns in table 4 and table 5 show how well the classical, 6-constant theory fits the accurate results obtained in the kilocycle per second range. The standard deviation calculated from either set of differences is 0.3 percent of the average value of 1/*E* or 1/*G*, respectively. The worst disagreement for either 1/*E* or 1/*G* is only 0.6 percent. It is evident that the classical theory of elasticity gives a very accurate representation of the elastic behavior in the kilocycle per second range.

## 9. Summary

1. The complete set of elastic constants and compliances for sapphire calculated from resonance measurements in the kilocycle per second frequency range is given.
2. Less accurate pulse velocity measurements in megacycle per second range indicate little, if any, frequency dependence of the elastic constants.
3. The values of *c*_11_ and *c*_33_ agree well with the results of the most accurate previous measurements, but the remaining constants and all of the compliances are in serious disagreement.
4. Values of Young’s modulus and the shear modulus for polycrystalline alumina calculated from the compliances are in good agreement with experimental values.
5. Curves for estimating Young’s modulus and the shear modulus for single crystal sapphire of any orientation are given.

## Figures and Tables

**Table 1:** Elasticity matrices

General elastic constant matrix					
*c*_11_	*c*_12_	*c*_13_	*c*_14_	*c*_15_	*c*_16_
*c*_12_	*c*_22_	*c*_23_	*c*_24_	*c*_25_	*c*_26_
*c*_13_	*c*_23_	*c*_33_	*c*_34_	*c*_35_	*c*_36_
*c*_14_	*c*_24_	*c*_34_	*c*_44_	*c*_45_	*c*_46_
*c*_15_	*c*_25_	*c*_35_	*c*_45_	*c*_55_	*c*_50_
*c*_16_	*c*_26_	*c*_36_	*c*_46_	*c*_56_	*c*_66_

Elastic constant matrix for sapphire referred to the coordinate system *x*_1_*x*_2_*x*_3_					

*c*_11_	*c*_12_	*c*_13_	*c*_14_	0	0
*c*_12_	*c*_11_	*c*_13_	−*c*_14_	0	0
*c*_13_	*c*_13_	*c*_33_	0	0	0
*c*_14_	−*c*_14_	0	*c*_44_	0	0
0	0	0	0	*c*_44_	*c*_14_
0	0	0	0	*c*_14_	½ (*c*_11_−*c*_12_)

Elastic compliance matrix for sapphire referred to the coordinate system *x*_1_*x*_2_*x*_3_					

*s*_11_	*s*_12_	*s*_13_	*s*_14_	0	0
*s*_12_	*s*_11_	*s*_13_	−*s*_14_	0	0
*s*_13_	*s*_13_	*s*_33_	0	0	0
*s*_14_	−*s*_14_	0	*S*_44_	0	0
0	0	0	0	*s*_44_	2*s*_14_
0	0	0	0	2s_14_	2(*s*_11_−*s*_12_)

**Table 2:** Dimensions and orientation of specimens

Specimen	Mass a	Nominal diameter	Actual length	Surface condition	*θ*	*ϕ*

	*g*	*in.*	*cm*			
SR 1	2.9876	0.100	14.700	f.p	13.6°	+13.0°
SR 2	3.1008	.100	15.244	f.p	10.7	+17.0
SR 3	3.0737	.100	15.224	f.p	65.4	−2.8
SR 4	3.0767	.100	15.232	f.p	29.4	−24.0
SR 5	2.1516	.100	10.664	f.p	45.6	+2.0
SR 6	3.0647	.100	15.188	f.p	41.2	+2.0
SR 7	3.0872	.100	15.218	f.p	67.8	−8.0
SR 8	3.0902	.100	15.258	f.p	12.5	+20.0
SR 9	3.0636	.100	15.240	f.p	89.0	−0.5
SR 10	1.9297	.100	9.591	f.p	88.6	+2.1
SR 11	6.9436	.150	15.240	f.p	59.5	−2.0
SR 12	6.9374	.150	15.282	c.g	36.2	+23.0
SR 13	6.9610	.150	15.280	c.g	11.9	+27.0
SR 14	6.9192	.150	15.268	c.g	3.0	−30.0
SR 15	6.9099	.150	15.288	c.g	18.8	+11.0
SR 16	6.9151	.150	15.212	c.g	45.0	−29.0
SR 17	6.9334	.150	15.244	c.g	40.7	−28.3
SR 18	6.9219	.150	15.268	c.g	59.6	−28.0
SR 19	6.9231	.150	15.280	c.g	64.0	−1.0
SR 20	4.7551	.125	15.260	c.g	43.5	−1.0
SR 21	4.7527	.125	15.228	c.g	48.8	−0.5
SR 22	3.1152	.100	15.292	f.p	89.0	+3.0
SR 23	3.1115	.100	15.282	f.p	89.5	−3.5
SR 24	3.1024	.100	15.246	f.p	89.5	−2.8
SR 25	3.1127	.100	15.300	f.p	89.0	−3.6
SR 26	3.1154	.100	15.296	f.p	90.0	−3.7
SR 27	3.1155	.100	15.300	f.p	88.8	−2.5
SR 28	3.1121	.100	15.296	f.p	89.5	−3.0
SR 29	3.1173	.100	15.316	f.p	89.0	−3.0

a Corrected for air buoyancy.

**Note:** c.g. means centerless-ground, f.p. means flame polished after centerless grinding. The nominal diameters were not used in calculations. Instead, the average radius was computed from the actual length, mass, and density, *ρ*=3.9860±0.0010 g/cm^3^.

**Table 3:** Resonance frequencies of sapphire rods

Specimen	Flexural a		Longitudinal	Torsional
	*f*_1_	*f*_2_		

SR 1	1100.1	1100.7	35673	20850
SR 2	1032.5	1033.7	34739	19940
SR 3	971.5	972.6	32747	21033
SR 4	990.0	990.9	33397	20547
SR 5	1908.9	1907.3	45060	…………..
SR 6	945.1	943.6	31746	21600
SR 7	998.3	997.5	33538	20616
SR 8	1021.9	1023.7	34464	20037
SR 9	993.8	1010.3	33868	20505
SR 10	2518.0	2539.3	53817	…………..
SR 11	1431.9	1432.7	32200	21210
SR 12	1378.7	1380.1	31145	21763
SR 13	1536.0	1537.0	34607	19943
SR 14	1557.1	…………..	35175	19704
SR 15	1487.6	1488.4	33703	20346
SR 16	1483.0	1484.1	33336	20596
SR 17	1472.5	1473.5	33160	20587
SR 18	1508.0	1507.7	34068	20152
SR 19	1433.7	1434.1	32420	20992
SR 20	1166.7	1169.2	31778	21331
SR 21	1172.7	1174.9	31819	21436
SR 22	1015.4	989.0	33727	20251
SR 23	1015.9	992.2	33786	20247
SR 24	1022.6	999.1	33871	20332
SR 25	1015.0	989.9	33768	20236
SR 26	1013.5	989.2	33735	20230
SR 27	1007.7	996.0	33757	20219
SR 28	1013.1	986.3	33715	20233
SR 29	1010.6	995.6	33790	20240

a The two flexural frequencies correspond to vibrations at right angles. The average value was used for calculations. All values are in cycles per second.

**Table 4:** Reciprocal Young’s modulus of sapphire All values are in units of 10^−13^ cm^2^/dyne. The standard deviation for 1*/E*, computed using the tabulated differences is ±0.0052 ×10^−13^ cm^2^/dyne.

Specimen	1/*E_f_* Flexural	1/*E_f_* Longitudinal	1/*E_f_* Calculated a	Difference b

SR 1	2.2764	2.2805	2.2759	0.0046
SR 2	2.2392	2.2366	2.2369	−.0003
SR 3	2.5242	2.5233	2.5193	.0040
SR 4	2.4262	2.4237	2.4277	−.0040
SR 5	2.7124	2.7167	2.7119	.0048
SR 6	2.6968	2.6976	2.6876	.0100
SR 7	2.4088	2.4079	2.4147	−.0068
SR 8	2.2684	2.2681	2.2621	.0060
SR 9	2.3530	2.3540	2.3529	.0011
SR 10	2.3543	2.3540	2.3564	−.0024
SR 11	2.6020	2.6048	2.5967	.0081
SR 12	2.7684	2.7685	2.7708	−.0023
SR 13	2.2505	2.2461	2.2545	−.0084
SR 14	2.1795	2.1744	2.1744	.0000
SR 15	2.3679	2.3624	2.3632	−.0008
SR 16	2.4414	2.4390	2.4404	−.0014
SR 17	2.4570	2.4546	2.4567	−.0021
SR 18	2.3214	2.3180	2.3125	.0055
SR 19	2.5564	2.5556	2.5645	−.0089
SR 20	2.6689	2.6674	2.6670	.0004
SR 21	2.6709	2.6717	2.6826	−.0109
SR 22	2.3537	2.3579	2.3560	.0019
SR 23	2.3488	2.3527	2.3515	.0012
SR 24	2.3424	2.3521	2.3518	.0003
SR 25	2.3440	2.3497	2.3502	−.0005
SR 26	2.3535	2.3554	2.3529	.0025
SR 27	2.3482	2.3512	2.3509	.0003
SR 28	2.3436	2.3477	2.3517	−.0040
SR 29	2.3486	2.3521	2.3507	.0014

a Calculated from eq (7) using the data of table 6.

b Difference=longitudinal value minus calculated value.

**Table 5:** Reciprocal shear modulus of sapphire All values are in units of 10^−13^ cm^2^/dyne. The standard deviation for 1*/G_f_* calculated from the difference column is ±.0178×10^−13^ cm^2^/dvne.

Specimen	$\frac{{\left(s_{34}^{\prime }\right)}^{2}+{\left(s_{35}^{\prime }\right)}^{2}}{2s_{33}^{\prime }}$	1*/G_f_* Torsional	1*/G_f_* Calculated	Difference

SR 1	0.0421	6.7186	6.7296	−0.0110
SR 2	.0291	6.8175	6.8066	.0109
SR 3	.0716	6.1888	6.2242	−.0354
SR 4	.0124	6.4156	6.4294	−.0138
SR 5^a^	………………..	………………..	………………..	………………..
SR 6	.0521	5.8792	5.9099	−.0307
SR 7	.0564	6.4282	6.4295	−.0013
SR 8	.0390	6.7491	6.7569	−.0078
SR 9	.0002	6.5208	6.5396	−.0188
SR 10 a	………………..	………………..	………………..	………………..
SR 11	.0734	6.0765	6.0763	.0002
SR 12	.0664	5.7365	5.7468	−.0103
SR 13	.0371	6.8003	6.7718	.0285
SR 14	.0020	6.9320	6.9302	.0018
SR 15	.0643	6.5469	6.5571	−.0102
SR 16	.0049	6.3939	6.3990	−.0051
SR 17	.0010	6.3692	6.3690	.0002
SR 18	.0189	6.6439	6.6407	.0032
SR 19	.0748	6.1704	6.1355	.0349
SR 20	.0506	5.9698	5.9498	.0200
SR 21	.0564	5.9428	5.9153	.0275
SR 22	.0024	6.5421	6.5334	.0087
SR 23	.0014	6.5528	6.5425	.0103
SR 24	.0008	6.5282	6.5419	−.0137
SR 25	.0010	6.5439	6.5451	−.0012
SR 26	.0019	6.5520	6.5396	.0124
SR 27	.0004	6.5544	6.5435	.0109
SR 28	.0009	6.5445	6.5421	.0024
SR 29	.0007	6.5319	6.5440	−.0121

a Too short for use.

**Table 6:** Best estimates of some compliances

Compliance	Value	Standard deviation

Calculated from Young’s modulus values a		

*s*_11_	2.3529×10^−13^cm^2^/dyne	0.0016×10^−13^cm^2^/dyne
*s*_33_	2.1694	.0024
*s*_44_+2*s*_13_	6.2183	.0088
*s*_14_	0.4901	.0057

Calculated from shear modulus values b		

*s*_11_–*s*_12_	3.0696×10^−13^cm^2^/dyne	0.0067×10^−13^cm^2^/dyne
*s*_44_	6.9400	.0084
*s*_11_+*s*_33_–(2*s*_13_+*s*_44_)	−1.6539	.0197
*s*_14_	0.4868	.0101

a Calculated by fitting eq (7) to Young’s modulus values calculated from longitudinal resonance frequencies.

b Calculated by fitting eq (8) to shear modulus values.

**Table 7:** Elastic compliances and constants of sapphire All *s_ij_* values in units of 10^−13^cm^2^/dyne. All *c_ij_* values in units of 10^12^dyne/cm^2^.

	Sundara Rao	Bhimasenachar	Mayer & Hiedemann	Present work

*s*_11_	2.84	2.32	2.18	2.353±0.002
*s*_33_	2.21	1.93	2.02	2.170±.002
*s*_44_	5.47	5.77	5.04	6.940±.008
*s*_12_	−0.95	−1.05	−0.50	−0.716±.007
*s*_13_	−.47	−0.38	−.16	−.364±.006
*s*_14_	−1.52	−1.71	−.49	.489±.005
*c*_11_	4.66	4.65	4.96	4.968±0.018
*c*_33_	5.06	5.63	5.02	4.981±.014
*c*_44_	2.35	2.33	2.06	1.474±.002
*c*_12_	1.27	1.24	1.09	1.636±.018
*c*_13_	1.17	1.17	0.48	1.109±.022
*c*_14_	0.94	1.01	.38	−0.235±.003

**Table 8:** Velocity of sound in sapphire All velocities in 10^3^cm/sec.

Direction a	Mode b	Calculated velocity	Measured velocity	Percent difference

Specimen SB1				

*X*	*L*	1117	1092	−2.2
	*T* pol *Y*	667	669	+0.3
	*T* pol Z	585	579	−1.0
*Y*	*L*	1118	1116	−0.2
	*T* pol *X*	651	645	−0.9
	*T* pol Z	600	609	+1.5
*Z*	*L*	1118	1100	−1.7
	*T*	608	594	−2.3

Specimen SB2				

*X*	*L*	1116	1088	−2.5
	*T* pol *Y*	676	662	−2.1
	*T* pol *Z*	575	564	−1.9
45° *YZ*	*L*	1095	1146	+4.7
	*T* pol *X*	581	609	+4.0
	*T* other	691	710	+2.7
	pol			
135° *YZ*	*L*	1041	1040	−0.1
	*T* pol *X*	671	659	−1.8
	*T* other	690	687	−0.4
	pol			

a The present directions are the actual face normals shown in figure 8.

b *“L”* means longitudinal, “ *T* pol *Y”* means transverse with the direction of vibration along *Y*, and “ *T* other pol” means transverse with the direction of vibration perpendicular to the other two modes.

**Table 9:** Comparison of calculated and measured elastic moduli for polycrystalline alumina All values in kilobars.

		Young’s modulus	Shear modulus
		
Measured (Lang)		4012	1591
Present data	Reuss theory	3973	1606
	Voigt theory	4084	1660
Mayer & Hiedemann	Reuss theory	4554	1996
	Voigt theory	4682	2070

**Table 10:** Comparison of compressibilities calculated from elastic compliances with values measured directly All values in 10^−13^ cm^2^ dyne.

	Linear compressibility		Volume compressibility
	Parallel to *x*_3_	Perpendicular to *x*_3_	
	
	Calculated from compliances		
	
Present data, 25° C	1.442	1.273	3.988
Mayer and Hiedemann, 27° C	1.70	1.52	4.74
	
	Measured		
	
Bridgman, 30° C, as reported	0.40	1.13	3.23
Bridgman, 30° C, interpreted	1.35	1.13	3.6
Madelung and Fuchs, 0° C	……….	……….	3.8

## Footnotes

1 Corundum is pure aluminum oxide, Al_2_O_3_, in the stable, *α* phase. The term sapphire is used synonymously in this paper.

2 A generalized theory, due to Laval and Raman, requires 10 constants and is discussed later.

3 Figures in brackets indicate the literature references at the end of this paper.

4 In a private communication dated March 28, 1960, R. H. Truell (Brown University) gives as tentative results from his joint investigation with P. H. Levy (Brookhaven National Laboratory) using a pulse velocity technique, C_33_=5.013×10^12^ dynes/cm^2^ and C_44_ = 1.478×10^12^ eynes/cm^2^. These values are seen to be in good agreement with the results presented here.
